# Photoelectrons Mediating Angiogenesis and Immunotherapy through Heterojunction Film for Noninvasive Disinfection

**DOI:** 10.1002/advs.202000023

**Published:** 2020-07-25

**Authors:** Yuan Li, Xiaomo Xu, Xiangmei Liu, Bo Li, Yong Han, Yufeng Zheng, Da‐fu Chen, Kelvin Wai Kwok Yeung, Zhenduo Cui, Zhaoyang Li, Yanqin Liang, Shengli Zhu, Xianbao Wang, Shuilin Wu

**Affiliations:** ^1^ The Key Laboratory of Advanced Ceramics and Machining Technology by the Ministry of Education of China School of Materials Science and Engineering Tianjin University Tianjin 300072 China; ^2^ Hubei Key Laboratory of Polymer Materials Ministry‐of‐Education Key Laboratory for the Green Preparation and Application of Functional Materials School of Materials Science and Engineering Hubei University Wuhan 430062 China; ^3^ State Key Laboratory for Mechanical Behavior of Materials School of Materials Science and Engineering Xi'an Jiaotong University Xi'an Shaanxi 710049 China; ^4^ State Key Laboratory for Turbulence and Complex System Department of Materials Science and Engineering College of Engineering Peking University Beijing 100871 China; ^5^ Laboratory Bone Tissue Engineering Beijing Research Institute Orthopaedics and Traumatology Beijing JiShuiTan Hospital Beijing 100035 P. R. China; ^6^ Department of Orthopaedics and Traumatology Li Ka Shing Faculty of Medicine The University of Hong Kong Pokfulam Hong Kong 999077 China

**Keywords:** angiogenesis, disinfection, heterojunctions, immunotherapy, photoelectrons

## Abstract

A light‐inspired hydroxyapatite (Hap)/nitrogen‐doped carbon dots (NCDs) modified graphene oxide (GO) heterojunction film is developed, which shows a promoted separation of interfacial electrons and holes and an inhibited recombination efficiency via hole depletion. The metabolism of bacteria on this film is significantly inhibited under light irradiation, due to the enhanced photocatalytic and photothermal effects. In addition, the electron transfer from the plasmonic membrane to the GO/NCD/Hap film further inhibits the adenosine triphosphate process of bacteria, thus leading to the synergetic antibacterial efficacy. Meanwhile, the electron transfer between film and cell membrane induces the Ca^2+^ flow after irradiation, which can promote the migration and proliferation of cells and alkaline phosphatase enhancement, thus favoring the tissue reconstruction. An in vivo test discloses that the vascular injury repair is achieved through the Ca^2+^‐activated PLC*γ*1/ERK pathway, identified by the enhanced CD31 expression. Moreover, the increased CD4^+^/CD8^+^ lymphocytes are ameliorative by activating the PI3K/P‐AKT pathway. Consequently, the electron transfer boosts the synergic photodynamic and photothermal therapeutic effects for bacterial infection by Ca^2+^ flow for immunotherapy. This mild phototherapy approach with GO/NCDs/Hap, which can simultaneously repair injured vessels and relieve inflammation reactions, will increase the clinical application of noninvasive phototherapy in the near future.

## Graphical abstract

The electron transfer between the Ti film and cell membrane induces the Ca^2+^ flow after irradiation, which can promote the migration and proliferation of cells and alkaline phosphatase enhancement, thus favoring the tissue reconstruction. An in vivo test discloses that the vascular injury repair is achieved through the Ca^2+^‐activated PLC*γ*1/ERK pathway, which is indicated by the enhanced CD31 expression.

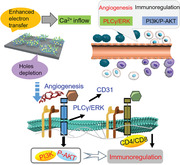

## 1 Introduction

Recently, phototherapy has been gradually used as an alternative antibiotic therapy due to the increasing bacterial tolerance.^[^ ^1^ ^]^ More multidrug‐resistant bacteria have been generated, such as carbapenem‐resistant Enterobacteriaceae and methicillin‐resistant *Staphylococcus aureus*.^[^ ^2^ ^]^ Different from traditional antibiotic therapies, phototherapy does not lead to homologous drug resistance due to a temporary short‐acting period.^[^ ^3^ ^]^ Generally, photodynamic therapy (PDT) and photothermal therapy (PTT) are used for tissue disinfection due to their penetrability, controllability, and excellent antibacterial activities.^[^ ^4^ ^]^ The produced reactive oxygen species (ROS), including •OH, ^1^O_2_, and •O_2_^−^, and simultaneous photothermia can affect the activities of proteins and RNA/DNA, adenosine triphosphate (ATP), and membrane structures.^[^ ^5^ ^]^ Through the combination of PDT and PTT, phototherapy can achieve a high and quick disinfection efficiency.^[^ ^6^ ^]^

However, simultaneous phototherapy tissue injuries (PTIs) can occur in normal cells and tissues during this course.^[^ ^7^ ^]^ Meanwhile, recent studies have reported that phototoxicity can cause side effects to vessel and cause concurrent inflammations after phototherapy.^[^ ^8^ ^]^ Because of the quite strong absorption toward the near‐infrared (NIR) window by melanin in the skin, the PTIs effects will be more intensive during skin phototherapy.^[^ ^9^ ^]^ In addition, injured tissues, including collagen aging, vascular malformation, and inflammation response under frequent or enduring irradiation can occur.^[^ ^10^ ^]^ However, the specific mechanism between phototherapy and injured vessels and immunoreaction is still unknown. Meanwhile, the method to solve the issue of PTIs effects for a safe and promising phototherapy has not been utilized.

Hydroxyapatite (Hap), a kind of traditional bioactive material, was used as a coating on implants due to its good thermostability, biodegradability, and osteogenesis.^[^ ^11^ ^]^ However, the application of this material for a photo‐responsive smart coating has not been studied due to low NIR light utilization.^[^ ^12^ ^]^ Some studies have reported the photocatalysis of Hap in an ultraviolet (UV) window, which is unsuitable for biomedical engineering.^[^ ^13^ ^]^

Carbon materials, such as nitrogen‐doped carbon dots (NCDs) and graphene oxide (GO), also act as excellent electron donors or receptors during photocatalysis.^[^ ^14^ ^]^ NCDs also have good applications, including biosensing and imaging, because of their outstanding upconversion and photoluminescence (PL) properties.^[^ ^15^ ^]^ Moreover, the fast recombination of electron–hole pairs inhibit the application of nano‐photocatalysis material for photocatalysis and phototherapy.^[^ ^16^ ^]^ Therefore, the modification of NCDs is necessary for enhancing the photocatalytic properties of these photocatalysts, accelerating the electron transfer, and inhibiting combination of electron–hole pairs.

**Scheme 1:**
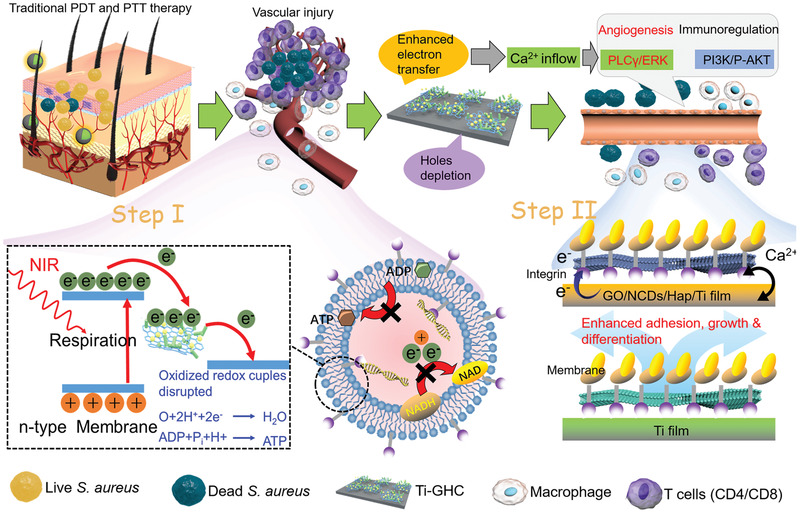
The mechanism of mild phototherapy using a GO/NCD/Hap film, which can repair injured vessels and simultaneously relieve inflammation reaction, thus promising a safe and noninvasive phototherapy in the near future.

Here, we constructed a NCDs/Hap modified GO heterojunction film for a mild phototherapy nanoplatform, which can not only kill up to 98.9% *S. aureus* but also repair injured vessels and simultaneously relieve inflammation by activating the PLC*γ*1/ERK and PI3K/P‐AKT pathways boosted by photoelectrons (**Scheme** **1**). The photocatalysis of the GO/NCD/Hap/titanium (Ti) film was drastically enhanced because of the increased electron–hole pair separation as a result of the boosted electron transfer between GO or Hap, and the depletion of holes by PO_4_^3−^ as well as OH^−^ from Hap. Furthermore, the electron transfer between the Ti substrate and plasmalemma assisted the phototherapy for an efficient and mild disinfection. The consequent photocurrent led to an obvious Ca^2+^ flow for cell adhesion and migration and tissue reconstruction. During the phototherapy evaluation in vivo, the original injured vessels were repaired by activating the PLC*γ*1/ERK pathway and regulated through a light‐induced Ca^2+^ flow. Meanwhile, the enhanced inflammation with respect to the induced CD4^+^/CD8^+^ lymphocytes and M1 polarization of IL‐6 and TNF‐*α* upregulation was relieved from the injured tissues by the PI3K/P‐AKT pathway. All in all, we provided one way for mild phototherapy using a GO/NCD/Hap film, which can repair injured vessels and simultaneously relieve inflammation reaction, thus promising a safe and noninvasive phototherapy in the near future.

## 2 Results and Discussion

### 2.1 Morphology and Structure Characterization

**Figure 1:**
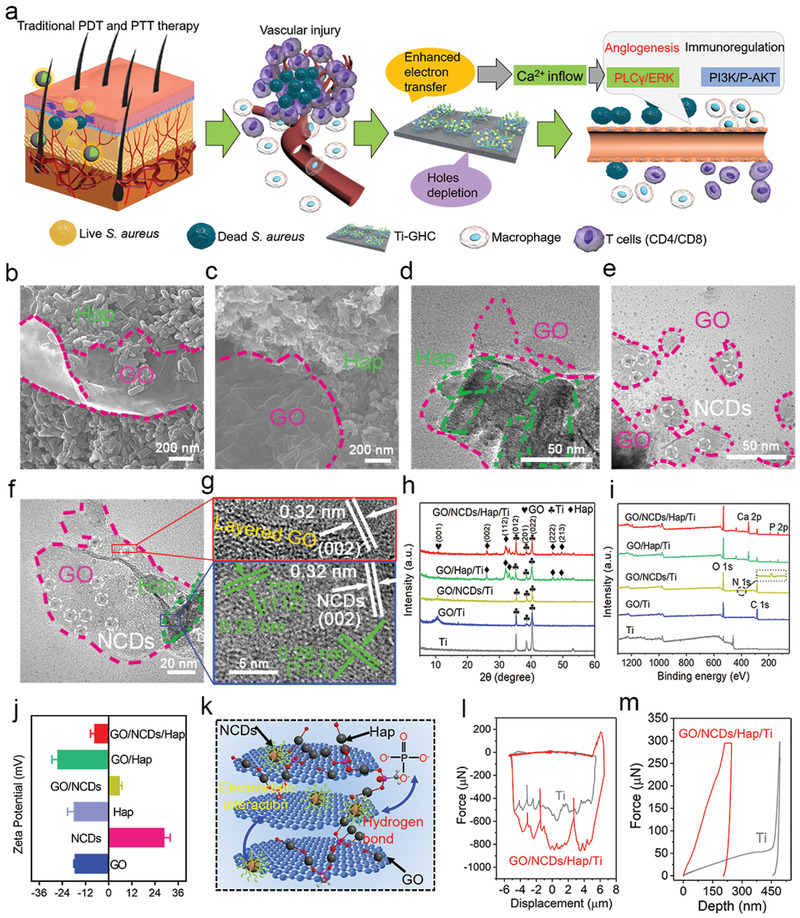
a,b,c) The SEM images of GO/Hap/Ti and GO/NCDs/Hap/Ti. d–f) The TEM images of GO/NCDs/Ti, GO/Hap/Ti, and GO/NCDs/Hap/Ti, respectively. g) The HRTEM image of GO/NCDs/Hap/Ti. h) The XRD curves of Ti, GO/Ti, GO/NCDs/Ti, GO/Hap/Ti, and GO/NCDs/Hap/Ti, respectively. i) The XPS curves of Ti, GO/Ti, GO/NCDs/Ti, GO/Hap/Ti, and GO/NCDs/Hap/Ti, respectively. j) Zeta potential values of Ti, GO/Ti, GO/NCDs/Ti, GO/Hap/Ti, and GO/NCDs/Hap/Ti, respectively. k) The structure sketch map and binding forces of GO/NCDs/Hap/Ti. l,m) The nanoscratch test and nanoindentation test of Ti and GO/NCDs/Hap/Ti.

The scanning electron microscopy (SEM) morphologies of the GO/NCD/Ti, GO/Hap/Ti, and GO/NCD/Hap/Ti films are shown in Figure S1, Supporting Information, **Figure** **1**a,b,c, respectively. Clearly, the lamellar GO and rod‐like Hap were combined together from GO/Hap/Ti and GO/NCDs/Hap/Ti. To prove the existence of loaded NCDs, the corresponding transmission electron microscopy (TEM) morphologies of the GO/Hap/Ti (Figure 1d), GO/NCD/Ti (Figure 1e), and GO/NCD/Hap/Ti (Figure 1f) films were further surveyed. An obvious integration was observed among them, and the uniform NCDs were distributed on the GO and Hap. As shown in the corresponding EDS measurement (Figure S2a, Supporting Information), the emerging N and C elements indicated the existence of NCDs. Meanwhile, we found the emerging Ca and P elements in the EDS results of GO/Hap/Ti and GO/NCDs/Hap/Ti (Figure S2b–c, Supporting Information), which represents the combination of Hap in the film. Furthermore, a clear lattice fringe and interfacial contact of GO, NCDs, and Hap appeared in the HRTEM image (Figure 1g). The (0 0 2) crystal face was combined with the (1 1 2) crystal face of the Hap and layered GO. Then, we investigated the XRD patterns of GO/NCDs/Ti, GO/Hap/Ti, and GO/NCDs/Hap/Ti, which all showed a common diffraction peak at 12°, resulting from GO. By mixing NCDs, Hap, or both of them, the intensity decreased. Moreover, the lattice planes of Hap (002, 112, 222, and 213) appeared in both the GO/Hap/Ti and GO/NCDs/Hap/Ti (Figure 1h). The X‐ray photoelectron spectroscopy full spectra were determined to further verify the binding of Hap and NCDs in the GO sheets. The emerging peaks of C1s (BE 284.4 eV) and N1s (BE 401.0 eV) and the emerging Ca (BE 346.8 eV) and P (BE 132.7 eV) from GO/NCDs/Hap/Ti indicated the existence of C1s, N1s, Ca2p, and P2p as compared with GO/Ti, demonstrating the successful loading of NCDs and Hap (Figure 1i). As shown in Figure 1j, the zeta potential measurement showed that, after the neutralization of electropositive NCDs (28.3 mV), electronegative GO (‐17.1 mV), and Hap (‐17.6 mV), they could be combined closely through an electrostatic interaction. The structure of GO/NCDs/Hap/Ti is schematically shown in Figure 1k. Besides the electrostatic interaction, the hydrogen bond between P—OH in Hap and C=O in GO makes the structure more stable. The carboxyl of GO also interacted with the Ca^2+^ and P—OH bonding of Hap. The binding force and Young's modulus obtained by the nanoindentation and nano‐scratches are shown in Figure 1l,m. Compared with Ti, the GO/NCD/Hap/Ti heterojunction film possessed a stronger binding force (approximately 820 µN) and intensity. Then, the contact angles were detected to evaluate the biological activity of the GO/NCD/Hap/Ti film (Figure S3, Supporting Information). Because of the hydrophilicity of Hap, GO/Hap/Ti and GO/NCDs/Hap/Ti possessed approximately 11° and 9°, respectively, which facilitated the adhesion and proliferation of cells.^[^ ^17^ ^]^

### 2.2 Photodynamic and Photothermal Properties

**Figure 2:**
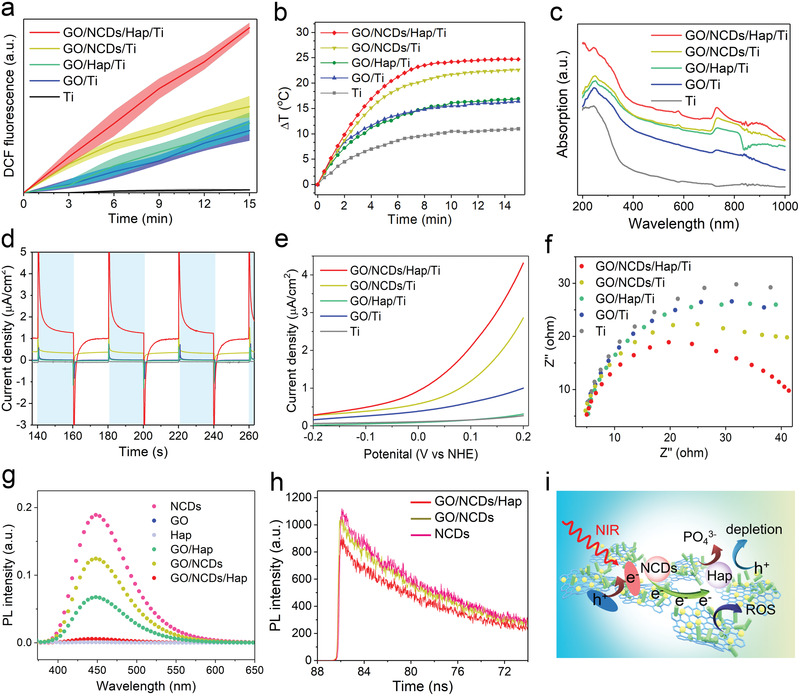
a) The ROS production of Ti, GO/Ti, GO/NCDs/Ti, GO/Hap/Ti, and GO/NCDs/Hap/Ti under NIR irradiation (0.5 W cm^−2^, 15 min). b) The photothermal curves of Ti, GO/Ti, GO/NCDs/Ti, GO/Hap/Ti, and GO/NCDs/Hap/Ti (0.5 W cm^−2^, 15 min). c) The UV–vis–NIR absorption curves of Ti, GO/Ti, GO/NCDs/Ti, GO/Hap/Ti, and GO/NCDs/Hap/Ti. d–f) The photocurrent curves, linear voltammetry sweep, and impedance values of Ti, GO/Ti, GO/NCDs/Ti, GO/Hap/Ti, and GO/NCDs/Hap/Ti. g) The PL spectra of NCDs, GO, Hap, GO, GO/NCDs, GO/Hap, and GO/NCDs/Hap. h) Corresponding fluorescent lifetime of NCDs, GO/NCDs, and GO/NCDs/Hap. i) The promoted separation of interfacial electrons and holes and inhibited recombination efficiency by and dissociated PO_4_^3−^.

To evaluate the antibacterial activity of GO/NCDs/Hap/Ti, the PDT and PTT performances were first tested. After 808 nm NIR light irradiation (0.5 W cm^−2^, 15 min), we found that GO/NCDs/Hap/Ti produced the most ROS yield amount, indicating its outstanding phototherapy capability (**Figure** **2**a). By contrast, GO/NCDs/Ti, GO/Hap/Ti, and GO/Ti induced less ROS yields because of the limited photoelectrons that participated in the photocatalysis. Meanwhile, GO/NCDs/Hap/Ti achieved the highest temperature rise of approximately 24.7 °C (Figure 2b) among all groups. The group of GO/NCDs/Ti possessed a better photothermal performance (22.6 °C) than GO/Hap/Ti (16.9 °C) and GO/Ti (16.4 °C). Then, the UV–vis–NIR spectra were carried out to analyze the photodynamic and photothermal performances (Figure 2c). A higher absorption under the NIR window (*λ* > 770 nm) and redshift absorption edge was shown, giving the possibility of a better PDT and PTT performances. To investigate the mechanism of ROS enhancement, the photocurrent and electrochemistry impedance were further carried out. The highest photocurrent density (1.23 µA cm^−2^) of GO/NCDs/Hap/Ti under four NIR on/off cycles was shown, and the varying sweep voltage indicated the fastest separation efficiency of electrons and holes due to the highest current density (3.28 µA cm^−2^) (Figure 2d–f).

Similarly, the minimum impedance appeared in GO/NCDs/Hap/Ti, illustrating the decrease of energy barrier and increase of photocatalysis reaction velocity, which led to a faster electron transfer during photocatalysis (Figure 2f). We also detected the separation efficiency of the electrons and holes of GO/NCDs/Hap/Ti with PL spectra and fluorescence lifetime by the time‐correlated single photon counting (Figure 2g,h). As shown, GO and Hap had a little generated fluorescence, distinct with NCDs. However, the recombination efficiency was greatly reduced and superior in GO/NCDs/Hap/Ti, which possessed the slowest fluorescent lifetime attenuation because of the enhanced electron transfer and depletion of h^+^ at the heterojunction interface. As shown in the enhanced photodynamic mechanism in Figure 2i, on the one hand, the photoelectrons and holes were produced due to the upconversion of NCDs distributed on the GO and Hap.^[^ ^18^ ^]^ Meanwhile, the interfacial electrons were transferred to the GO and Hap, which led to the enhanced electron–hole separation. On the other hand, the dissociated PO_4_^3−^ and OH^−^ from Hap accelerated the hole depletion through neutralization. The transferred electrons and dissipative h^+^ enhanced the separation, and the inhibited recombination of electron–hole pairs finally promoted the ROS production.

### 2.3 Antibacterial Activity and Mechanism Explanation

**Figure 3:**
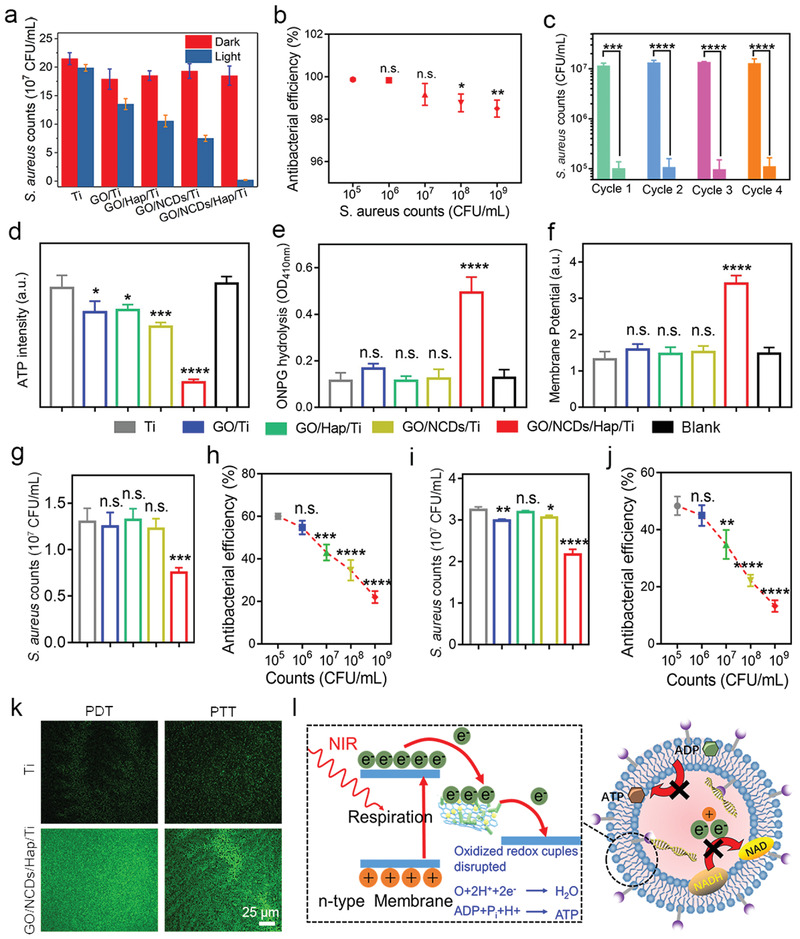
a) The antibacterial efficiency of Ti, GO/Ti, GO/NCDs/Ti, GO/Hap/Ti, and GO/NCDs/Hap/Ti under NIR irradiation (0.5 W cm^−2^, 15 min) toward *S. aureus*. b) The antibacterial efficiency of GO/NCDs/Hap/Ti at gradient CFU concentration of *S. aureus* under NIR irradiation (0.5 W cm^−2^, 15 min). c) Circulatory antibacterial efficiency of GO/NCDs/Hap/Ti with 4 cycles. d–f) The ATP intensity, ONPG hydrolysis degree, and membrane potential of Ti, GO/Ti, GO/NCDs/Ti, GO/Hap/Ti, and GO/NCDs/Hap/Ti under NIR irradiation (0.5 W cm^−2^, 15 min). g) The antibacterial efficiency of Ti, GO/Ti, GO/NCDs/Ti, GO/Hap/Ti, and GO/NCDs/Hap/Ti under PDT condition (water bath). h) The antibacterial efficiency of GO/NCDs/Hap/Ti at gradient CFU concentration of *S. aureus* under PDT condition. i) The antibacterial efficiency of Ti, GO/Ti, GO/NCDs/Ti, GO/Hap/Ti, and GO/NCDs/Hap/Ti under PDT condition (keep in corresponding temperature to photothermal curves). j) The antibacterial efficiency of GO/NCDs/Hap/Ti at gradient CFU concentration of *S. aureus* under PTT condition. k) The ROS staining toward *S. aureus* with DCFH‐DA probe of Ti and GO/NCDs/Hap/Ti. l) The antibacterial mechanism of GO/NCDs/Hap/Ti by electrons transfer boosted PDT/PTT combination disinfection.

*S. aureus* was employed to evaluate the specific antibacterial activity of GO/NCDs/Hap/Ti. After 808 nm light irradiation, approximately 98.9% antibacterial efficiency of GO/NCDs/Hap/Ti was shown, which is higher than that of GO/NCDs/Ti and GO/Hap/Ti, due to the sufficient ROS production and higher photothermal temperature of the former (**Figure** **3**a; Figure S4, Supporting Information). Regarding the gradient CFU concentration of *S. aureus*, a higher antibacterial efficiency of 99.9% was achieved at the least infective concentration of approximately 10^5^ CFU mL^−1^ (Figure 3b). Meanwhile, the antibacterial efficacy of GO/NCDs/Hap/Ti toward *Escherichia coli* was also as high as 99.8% (Figure S5, Supporting Information). With the increased cycles of antibacterial assays, a little depressed tendency of antibacterial efficiency occurred and the corresponding spread plates verified it (Figure 3c; Figures S6,S7, Supporting Information). Subsequently, the influence of PDT and PTT on the *S. aureus* membrane was detected through intimal permeability (ONPG hydrolysis activity), membrane potential, and ATP activity. Increased intimal permeability and membrane potential were shown in GO/NCDs/Hap/Ti compared with GO/NCDs/Ti and GO/Hap/Ti, which affected little to membrane state (Figure 3d,e). As a result, the biochemical reaction was greatly decreased to produce the necessary ATP metabolically (Figure 3f). Combined with the ROS effect, the photothermal effect greatly influenced the membrane activity, which was confirmed by the SEM morphology of *S. aureus* (Figure S8, Supporting Information), proving the various degrees of damage by PDT and PTT. Compared with the blank group (without any antibacterial material and method), we found that the Ti group possessed negligible antibacterial activity with the blank group based on the ONPG hydrolysis activity, membrane potential, and ATP activity data (Figure S9, Supporting Information).

However, single PDT and mild PTT did not lead to enough sterilization effects compared with the combined phototherapy. To further investigate the effect of individual PDT (water bath treatment) and PTT (constant temperature treatment) to *S. aureus*, an antibacterial test was performed, and the results are shown in Figure 3g–j. We found that a higher ROS and higher temperature resulted in a higher CFU reduction (Figure 3g,i). Moreover, with the decline of the CFU of *S. aureus*, a similar increase in antibacterial effects was shown (Figure 3h,j). To prove it, the ROS staining with a 2′,7′‐dichlorofluorescein diacetate (DCFH‐DA) probe was performed under PDT and PTT, as shown in Figure 3k. More intracellular ROS were induced after the NIR irradiation of GO/NCDs/Hap/Ti as compared with individual Ti, indicating the increased bacterial damage. As proven in a previous study, the biological redox potential values of a bacterial transmembrane protein complex in a respiration chain ranges from ‐4.1 to ‐4.8 eV, which is higher than the potential of a Ti film.^[^ ^19^ ^]^ As a result, the electrons on the bacterial membrane between the Ti film and bacteria boosted the interruption of the respiration chain and membrane structure damage. As a result, the membrane electrons were extracted from the transmembrane protein complex of *S. aureus*, which resulted in the discharge process of the respiratory chain. Then, the ROS was over‐produced and the ATP synthesis process was inhibited, which led to the final *S. aureus* starvation and death.

### 2.4 Cell Response and Biosecurity

**Figure 4:**
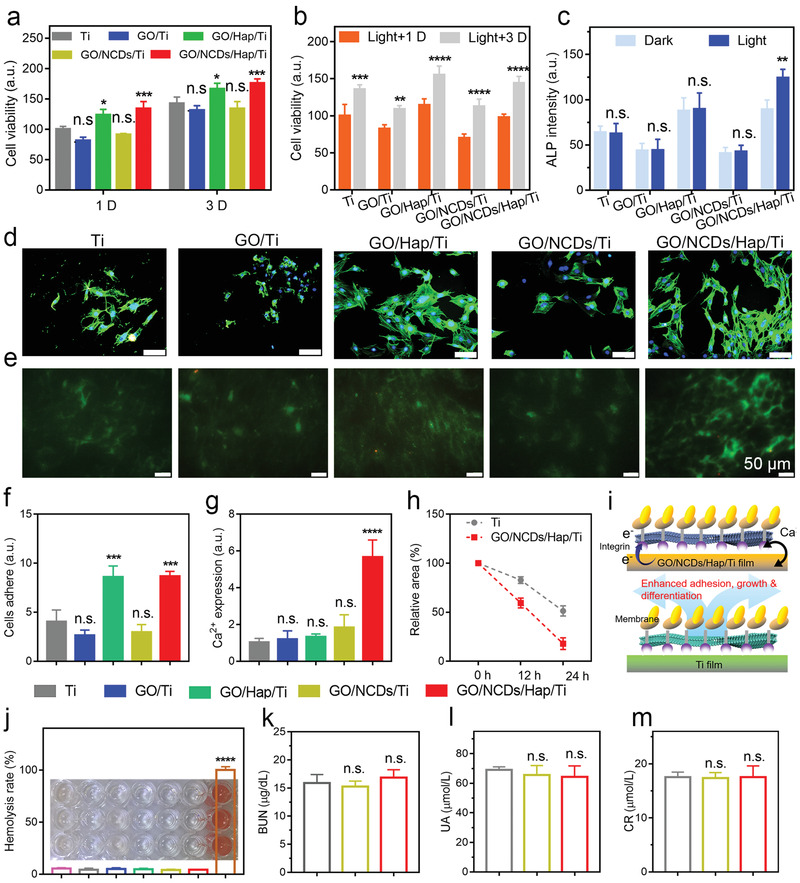
a) The cell viability of Ti, GO/Ti, GO/NCDs/Ti, GO/Hap/Ti, and GO/NCDs/Hap/Ti after 1 D and 3 D incubation. b) The cell viability of Ti, GO/Ti, GO/NCDs/Ti, GO/Hap/Ti, and GO/NCDs/Hap/Ti after NIR irradiation (0.5 W cm^−2^, 15 min). c) The ALP intensities of Ti, GO/Ti, GO/NCDs/Ti, GO/Hap/Ti, and GO/NCDs/Hap/Ti after 14 D incubation. d) The cells fluorescence staining with FITC (green fluorescence marked cytoplasm) and DAPI (green fluorescence marked cell nucleus). e) The Ca^2+^ expressions stained by Fluo‐3 AM probe after NIR irradiation. f) The cells adhesion intensities of Ti, GO/Ti, GO/NCDs/Ti, GO/Hap/Ti, and GO/NCDs/Hap/Ti after NIR irradiation. g) The Ca^2+^ expressions of Ti, GO/Ti, GO/NCDs/Ti, GO/Hap/Ti, and GO/NCDs/Hap/Ti after NIR irradiation, h) The cells migration rates of Ti and GO/NCDs/Hap/Ti after NIR irradiation of 0, 12, and 24 h. i) The corresponding mechanism of Ca^2+^ flow, which can promote the migration and proliferation as well as ALP enhancement for tissue reconstruction. j) The blood hemolysis rates of Ti, GO/Ti, GO/NCDs/Ti, GO/Hap/Ti, and GO/NCDs/Hap/Ti. k–m) The toxicity detection of BUN, CR, and UA of Ti, GO/NCDs/Ti, and GO/NCDs/Hap/Ti after light treatment.

MTT assay was first performed to reflect the cytotoxicity with or without NIR irradiation after 24 h (**Figure** **4**a,b). The cell viability in GO/Ti (81.4%) and GO/NCDs/Ti (91.1%) was lower than that in Ti because of the higher contact angle that was not beneficial for cell adhesion. We found that the cell viability of Ti group was similar to the one of the blank group, indicating negligible activity of promoting cell proliferation. By contrast, GO/Hap/Ti and GO/NCDs/Hap/Ti had outstanding cytocompatibility due to the good biocompatibility of Hap and smaller lower contact angle. Then, the ALP activity of the GO/NCD/Hap/Ti film for bone implantation function was detected (Figure 4c). After 14 days incubation, 1.43‐ and 1.61‐times enhancements of the ALP activity toward GO/Hap/Ti and GO/NCDs/Hap/Ti after the NIR irradiation were shown as compared with Ti, indicating their osteogenic capability. By contrast, an indistinctive value was shown for GO/Ti and GO/NCDs/Ti. The F‐actin/DAPI fluorescence staining was employed to analyze the cell adhesion and proliferation activities (Figure 4d). We can find the most extended filopodia and lamellipodia in the cells on GO/Hap/Ti and GO/NCDs/Hap/Ti, which revealed an excellent cell viability. Meanwhile, the cells on GO/NCDs/Hap/Ti achieved the most quantities, and proliferation indicated the best cells adhere state (Figure 4d,f). Because of the mechanism of the photoinduced electron flow between GO/NCDs/Hap/Ti and contacted cells, which can influence the Ca^2+^ flow,^[^ ^20^ ^]^ the Ca^2+^ staining with Fluo‐3 AM probe was performed after the NIR irradiation (Figure 4e). Obviously, GO/NCDs/Hap/Ti possessed the highest Ca^2+^ expression due to its maximum photocurrent value. GO/NCDs/Ti expressed lesser Ca^2+^ than GO/NCDs/Hap/Ti and more Ca^2+^ expression than GO/Hap/Ti and GO/Ti (Figure 4e,g), which was consistent with the photocurrent under the NIR irradiation (Figure 2d). Furthermore, the cell migration rate was evaluated through a scratch assay after the NIR irradiation and 24 h co‐culture in vitro (Figure 4h; Figure S10, Supporting Information). As shown, GO/NCDs/Hap/Ti promoted the fastest migration than GO/Hap/Ti, GO/NCDs/Ti, and GO/Ti due to its good biocompatibility and Ca^2+^ expression, which was closely related with the tissue reconstruction activity. The relevant mechanism is shown in Figure 4i. The GO/NCDs/Hap/Ti generated electrons and developed the photocurrent between GO/NCDs/Hap/Ti and cells, which led to the Ca^2+^ flow and accelerated the cell adhesion and differentiation. The hemolysis assay in vitro was performed to evaluate the security of GO/NCDs/Hap/Ti after a 4 h incubation with erythrocytes (Figure 4j). The hemolysis rates of GO/Hap/Ti, GO/NCDs/Ti, and GO/NCDs/Hap/Ti were lower than the international standard (5%) except of GO, which possessed minor hemolysis and is consistent with the viability.^[^ ^21^ ^]^ In addition, the biosafety of GO/NCDs/Hap/Ti was important in in vivo to tissues and organs. As shown in Figure 4k–m, the renal toxicity of blood urea nitrogen, uric acid, and creatinine was further detected. Compared with Ti, no clear difference was observed, indicating the little effect to renal. All in all, the results demonstrated the outstanding biosecurity of GO/NCDs/Hap/Ti in vitro and in vivo after the implantation.

### 2.5 Animal Tests In Vivo

**Figure 5:**
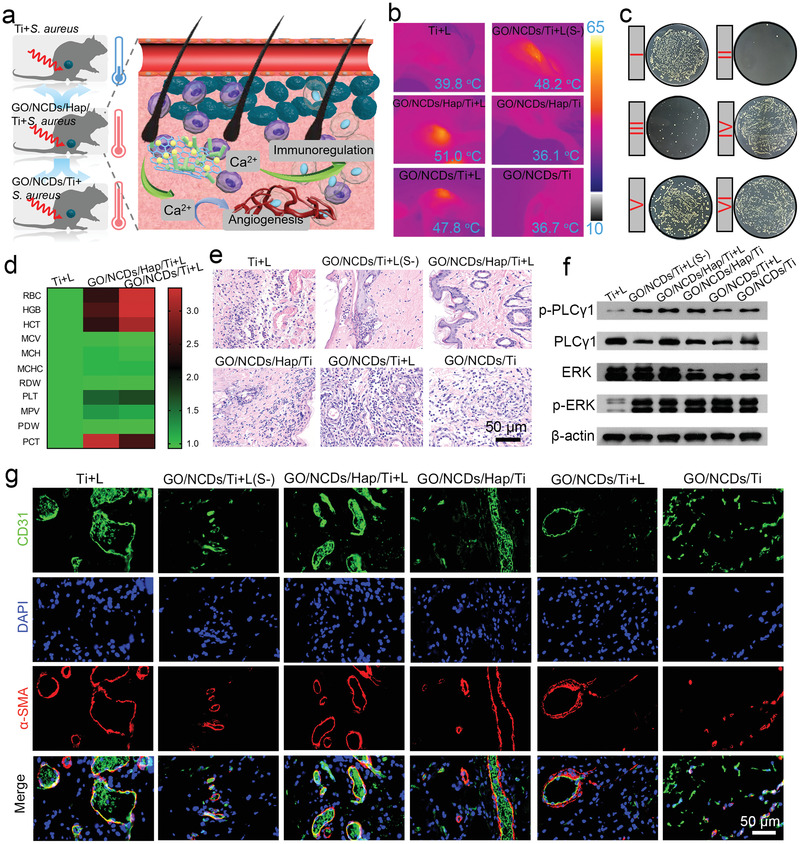
a) The schematic diagram of mild phototherapy through the NIR induced Ca^2+^ flow and then activated the PLC*γ*1/ERK and PI3K/P‐AKT pathway for injured vessel repair and inflammation relieve. b) The photothermal images in vivo after NIR irradiation (0.5 W cm^−2^, 15 min). c) The corresponding spread plates of *S. aureus* after NIR irradiation with (b). d) The blood routine examination of Ti+L, GO/NCDs/Ti+L, and GO/NCDs/Hap/Ti+L at 1 D treatment. e) The H&E staining of infected tissue near the implant at 1 D treatment. f) The WB analysis regarding to the vascular repair pathway at 3 D treatment. g) The immunofluorescent staining of CD31 (green), DAPI (blue), and *α*‐SMA (red) at 6 D treatment.

We further evaluated the mild PDT and PTT therapy efficiency toward *S. aureus* in vivo by implanting GO/NCDs/Hap/Ti at the tibia of rats (**Figure** **5**a). Meanwhile, the additional group of GO/NCDs/Ti+L(S‐) represents the treatment without *S. aureus* injection. Compared with the mild phototherapy moderated by the Ca^2+^ flow caused by GO/NCDs/Hap/Ti, the traditional phototherapy can injure partial blood vessels and lead to a collateral inflammatory response because of the resulting ROS and photothermia effects, which were unsuitable tissue regeneration. As shown in Figure 5b, the corresponding photothermal images of irradiated tissues showed 48.2 °C of GO/NCDs/Ti+L(S‐) (NIR light‐treated GO/NNCDs/Ti group) and 51.0 °C of GO/NCDs/Hap/Ti+L (NIR light‐treated GO/NCDs/Ti group), which indicated a good photothermal effect and the achieved penetrability of phototherapy. The spread plate results in Figure 5c disclose that after the primary irradiation toward GO/NCDs/Hap/Ti+L, over 98.1% of disinfection efficiency was achieved, whereas GO/NCDs/Ti+L possessed an insufficient efficiency of 55.4% (Figure S11, Supporting Information). The blood routine examination and main organ staining were carried out at 1 D (day), 2 D, and 3 D treatments with unobvious distinction, indicating the safety toward the blood and organs (Figure 5d; Figures S12,S13 and Table S1, Supporting Information). Meanwhile, hematoxylin–eosin (H&E) staining was used to reflect the inflammatory degree of tissues that was caused by phototherapy damage‐infected *S. aureus* (Figure 5e). We found that GO/NCDs/Hap/Ti+L had the least inflammation compared with GO/NCDs/Ti+L and Ti+L after the irradiation. After the 3 D treatment, the inflammation also appeared and became palliative toward GO/NCDs/Ti+L and GO/NCDs/Ti due to the recovery of tissues and immunity (Figure S14, Supporting Information). To get insights on the influence of repaired blood vessels, a Western blot (WB) assay was performed to evaluate the GO/NCD/Hap/Ti‐induced pathway regarding angiogenesis. In previous reports, the PLC*γ*1 from rats nullizygous was subject to embryonic lethality because of obviously impaired angiogenesis and erythrogenesis, and PLC*γ*1 is necessary toward the function of VEGF and arterial development.^[^ ^22^ ^]^ The PLC*γ*1 and ERK of GO/NCDs/Hap/Ti+L were expressed more compared with GO/NCDs/Ti and GO/NCDs/Hap/Ti. As we know, the phosphorylation of ERK (pERK) always occurs in endothelial cells undergoing angiogenesis. And the pERK in endothelial cells was ectopically induced by Vegfa, which contributed to the angiogenesis process.^[^ ^23^ ^]^ After the inhibition of expressions, the expressions were similar to GO/NCDs/Ti and GO/NCDs/Hap/Ti, indicating the photo‐induced Ca^2+^ flow and activated PLC*γ*1/ERK pathway (Figure 5f). Blood vessel staining was further performed to estimate the effects of GO/NCDs/Hap/Ti in mild phototherapy (Figure 5g). GO/NCDs/Ti+L caused the most serious injury among all groups because of the insufficient disinfection and toxicity in a normal phototherapy. However, GO/NCDs/Hap/Ti+L showed a clear CD31 expression, which is represented by the growing blood vessels after the light irradiation. Based on the results in immunohistochemical assay after light treatment, we found that GO/NCDs/Ti+L caused the most serious injury among all groups because of the insufficient disinfection effect and toxicity in a normal phototherapy (Figure S15, Supporting Information). However, GO/NCDs/Hap/Ti+L showed obvious CD31 expression which showed the recuperative blood vessels after the light irradiation.

**Figure 6:**
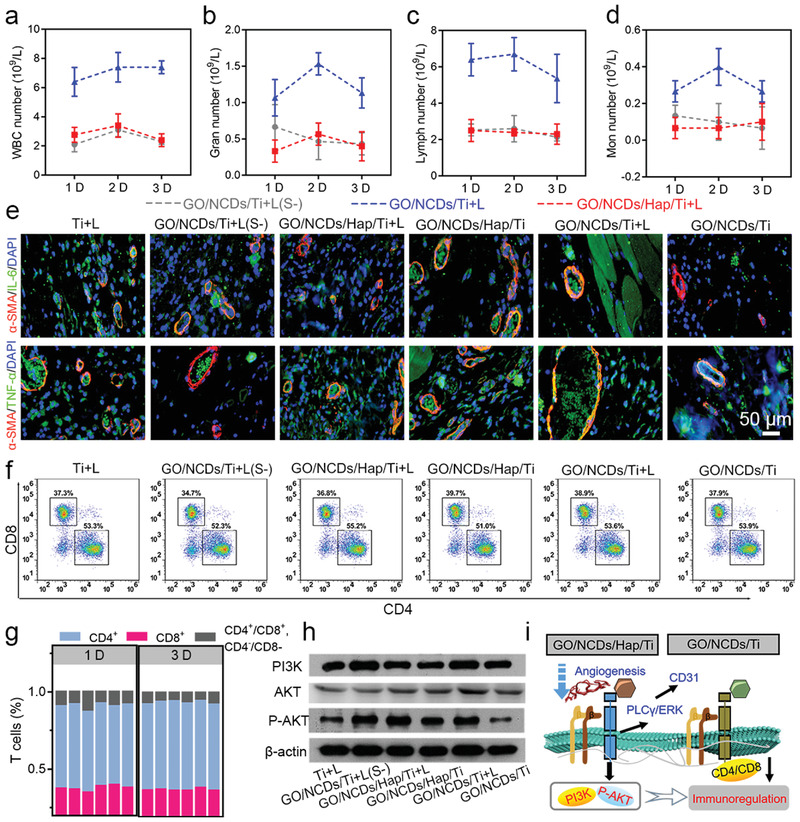
Immune cells number calculation in the blood at 1 D, 2 D, and 3 D treatment after NIR irradiation, a) the WBC, b) Gran, c) Lymph, and d) Mon (grey imaginary line indicated Ti+L, blue imaginary line indicated GO/NCDs/Ti+L, red imaginary line indicated GO/NCDs/Hap/Ti‐L). e) Immunofluorescent staining of IL‐6 and TNF‐*α* (green), DAPI (blue), and *α*‐SMA (red) after irradiation for 3 D. f) The flow cytometry of CD4 and CD8 lymphocyte in the tissue after irradiation for 3 D. g) The CD3 lymphocyte statistics of CD4^+^, CD8^+^, and CD4^+^/CD8^+^, CD4^−^/CD8^−^. h) The WB analysis regarding to the inflammation relieve at 3 D treatment. i) The corresponding mechanism of mild phototherapy mechanism with GO/NCDs/Hap/Ti film through activating PLC*γ*1/ERK and PI3K/P‐AKT pathway.

The resulting inflammation after the normal phototherapy was further analyzed. Specifically, the WBC, Gran, Lymph, and Mon numbers were counted from whole blood at 1 D to 3 D (**Figure** **6**a–d). Regarding GO/NCDs/Hap/Ti+L, the parameters were drastically decreased after the light treatment, indicating the relieved inflammation response. However, GO/NCDs/Ti+L had a stronger inflammation, consistent with the vascular injury. Furthermore, the specific inflammatory factors of IL‐6 and TNF‐*α* with respect to M1 polarization were evaluated. The obvious upregulation of GO/NCDs/Ti+L (strong green fluorescence) and downregulation of GO/NCDs/Hap/Ti+L (weak green fluorescence) are shown in Figure 6e. To get insights on the influence to T cells, the CD4 and CD8 cells were analyzed in the tissues after phototherapy (Figure 6f). GO/Ti possessed lower CD4 cells and more CD8 cells, which was serious after the irradiation as compared with GO/NCDs/Hap/Ti at 1 D, indicating a weak inflammation. After the 3 D treatment, GO/NCDs/Hap/Ti+L relieved the CD4^+^/CD8^+^ cell ratio as compared with GO/NCDs/Hap/Ti, indicating that the Ca^2+^ influenced the tissue inflammation and relieved it (Figure S16, Supporting Information). And the data about neutrophil granulocyte in Figure S17, Supporting Information, which reflected that GO/NCDs/Hap/Ti+L had the least inflammation compared with GO/NCDs/Ti+L and Ti+L after the irradiation (Figure S17, Supporting Information). Similarly, the damage in tissues was analyzed through CD4^+^/CD8^+^ T cell ratio of the tissues near GO/NCDs/Hap/Ti (Figure 6g). Generally, a large CD4^+^/CD8^+^ ratio indicated less inflammation, that is, GO/NCDs/Hap/Ti contributed to the minimum T cell response, which was beneficial for the tissue reconstruction. After the 3 D treatment, the tissue inflammation was decreased. Furthermore, the WB assay with respect to the tissue inflammation was investigated (Figure 6h). After the phototherapy treatment, GO/NCDs/Hap/Ti+L relieved the tissue injury as compared with GO/NCDs/Ti+L and GO/NCDs/Hap/Ti. The (PI3K)/pAKT pathway can be activated in the majority of human tumor and this signal path is known to play a significant role in most cellular functions including cells proliferation, adhesion, migration, metabolism, and survival as well as the tissue angiogenesis.^[^ ^24^ ^]^ The total AKT expression was not distinct between the groups with or without light irradiation. Compared with Ti+L, the overexpression of PI3K/P‐AKT in GO/NCDs/Hap/Ti+L indicated that the photocurrent‐induced Ca^2+^ flow inhibited the inflammatory response. As a result, the angiogenesis and immunoregulation were moderated by GO/NCDs/Hap/Ti through the Ca^2+^ flow between the cells and GO/NCDs/Hap/Ti during the phototherapy. The mechanism of the mild phototherapy mechanism is schematically shown in Figure 6i. During this process, the PLC*γ*1/ERK and PI3K/P‐AKT pathways were involved in the mild phototherapy, which is promising for low damages in tissues.

## 3 Conclusion

In this work, we constructed an N‐doped CDs/Hap modified GO film for mild PDT and PTT nanoplatforms, which can not only kill over 98.9% of *S. aureus* under 15 min NIR irradiation but also repair vessels and relieve inflammation through the PLC*γ*1/ERK and PI3K/P‐AKT pathways. The NCDs and Hap enhanced the photocatalysis of the GO/NCDs/Hap/Ti film because of the increased electron–hole pair separation by GO/Hap and the depletion of h^+^ by Hap. In addition, the electron transfer between the Ti film and plasmalemma boosted the PDT and PTT performances for an efficient disinfection. Meanwhile, the resulting light current led to an obvious Ca^2+^ flow for cell adhesion and migration and tissue reconstruction. Furthermore, we demonstrated that the injured vessels and enhanced inflammation can be repaired through the PLC*γ*1/ERK pathway and Ca^2+^ regulation. The serious inflammation with respect to the increased CD4^+^/CD8^+^ lymphocytes and M1 polarization of expressed IL‐6 and TNF‐*α* caused by phototherapy was relieved from the injured tissues through the PI3K/P‐AKT pathway. As a result, we developed a mild phototherapy using the GO/NCDs/Hap film with the biological functions of rapid in vivo disinfection, vessel repair, and inflammation reduction, which will be promising for a safe and noninvasive therapeutic strategy.

## 4 Experimental Section

##### Synthesis of GO/Hap Composites

Hap nanorods were prepared through a typical hydrothermal method. First, a 0.5 mol L^−1^ Ca(NO_3_)_2_·4H_2_O aqueous solution was mixed with a 0.3 mol L^−1^ (NH_4_)_2_HPO_4_ aqueous solution at a molar ratio of Ca:P = 1:1.67, and the pH value was adjusted to 8.7 with 25% ammonia water. The mixture reacted in a Teflon stainless steel kettle under 130 °C for 12 h. After the reaction, the precipitates were separated via a centrifugal separation, washed with deionized water for three times, and then freeze‐dried and calcined at 400 °C for 5 h. 1 g Hap powder obtained above was mixed with 1 mg mL^−1^ graphene oxide solution, and then Hap was loaded on the GO by ultrasonic for 24 h.

##### Synthesis of NCDs

2 g monohydrate citric acid and 0.62 g glycine were dissolved in 5 mL water, and then the mixture was put into the oven and dried at 70 °C for 12 h in a vacuum. The viscous slurry was obtained and transferred to a stainless‐steel reactor with a Teflon tank for 3 h at 200 °C. The final product was neutralized with 1 mol L^−1^ sodium hydroxide and diluted to 100 mL for reserve.

##### Preparation of GO/NCDs/Hap/Ti

The prepared titanium sheets were sequentially polished using a polishing machine at 240#, 400 #, 600#, 800#, and 1200# and then ultrasonically washed three times with deionized water and ethanol. The concentrated sulfuric acid was prepared as follows: concentrated hydrochloric acid and water with a ratio of 1:1 were mixed in an acid etching solution, and a titanium sheet was placed in the acid etching solution at 60 °C for 1 h to obtain a titanium sheet with active groups on the surface. The treated titanium sheet was spin‐coated with GO/Hap at 3000 rpm, dried, and then placed in a CVD atmosphere under a nitrogen atmosphere to be cured at 500 °C for 1 h to bond the coating to the substrate. Subsequently, the calcined titanium sheet was immersed in the carbon quantum dot solution prepared above and vacuum‐loaded for 24 h to obtain a sample.

##### Characterization

FE‐SEM (Sigma‐500, Sigma, Germany) was used to examine the sample morphology and EDS; XRD showed the composition and phase. Nanoindentation was employed to assess the adhesion strength between the coatings and substrates. UV–vis spectrophotometry was performed on UV‐3600 (Shimadzu, Japan), and TEM ( FEI, TF20, USA) was performed to examine the morphology. The cell morphology was observed under an inverted fluorescence microscope (IX73, Olympus, Japan).

##### ROS Yield Test

The production of reactive oxygen was tested with the DCFH‐DA probe. The samples (GO/Ti, GO/NCDs/Ti, GO/HAP/Ti, GO/NCDs/HAP/Ti) were placed in a 96‐well plate, followed by the preparation of 200 µL of the DCFH‐DA (10 µg mL^−1^) solution. The 808 nm laser was used to illuminate the samples in the well for 15 min at a power of 0.5 W cm^−1^, and the fluorescence value of the sample was measured every 3 min using a microplate reader, with an excitation wavelength of 488 nm and absorption wavelength of 525 nm. The reactive oxygen yield of the sample was determined by calculation. Three separate replicates were performed for each set of samples.

##### Photocurrent Experiment

The photocurrent properties of the samples were tested using a three‐electrode method. The platinum electrode was used as the counter electrode, the Ag/AgCl electrode was used as the reference electrode, and the working electrode was made of a titanium plate encapsulated with a silica gel. The electrolyte solution was a 0.1 m sodium sulfate solution. The samples were tested using a CHI‐660 electrochemical workstation, and the results were measured under an 808 nm laser. The linear sweep voltammetry scan rate is 10 mV s^−1^.

##### Antibacterial Experiment

The samples and *S. aureus* solution (200 µL, about 10^7^ CFU per mL) were placed in different numbers of samples into a 96‐well plate. 100 µL of the bacterial solution was added to the surface of the sample, and the illuminating group was irradiated with 808 nm near‐infrared light for 15 min to maintain the surface temperature of the titanium sheet at 50 °C.

##### Biocompatibility Test

The cells were incubated with GO/Ti, GO/NCDs/Ti, GO/HAP/Ti, and GO/NCDs/Hap/Ti for 1 D and 3 D for MTT evaluation and fluorescence staining with MC3T3‐E1 cells, respectively. After different incubation times, the cellular medium was extracted and incubated with an MTT solution (200 µL, 0.5 mg mL^−1^) for the next 4 h in a 37 °C incubator. Then, the MTT solution was extracted, and the DMSO solution was added. The OD value was detected after a 15 min shock at 600 nm. Regarding the MTT under light, the cells were irradiated for 15 min (0.5 W cm^−2^), and the corresponding cell viability was measured at OD_600_. Similarly, the cell fluorescence was carried out after the co‐incubation of the samples and cells for 1 D by staining with FITC and DAPI dye liquor (Beyotime, China). The cells were stood until dry for photograph. The cell migration test was performed using the scratch method in the 48‐well plate before the co‐incubation and stained and photographed after 24 h.

##### Ca^2+^ Response and Biosecurity

After the cells and samples were co‐cultured for 24 h and irradiated for 15 min, the medium was extracted and stained with Fluo‐3 AM (Yeasen, China) for 15 min. Then, the cells were irradiated and desiccated for observation of fluorescent images.

##### Animal Tests In Vivo

The antibacterial activity of the samples and vascular repair and inflammation relief capability were tested using 200 g rats purchased from Hubei Provincial Centers for Disease Control and Prevention, and all procedures were approved by the Department of Orthopedics of Union Hospital, Tongji Medical College, and Huazhong University of Science and Technology in Wuhan. The *S. aureus*‐treated rats were divided into six groups (with and without light treatment). Before the Ti implantation, 20 µL *S. aureus* were added to the surface and air‐dried for 20 min. Then, the rats were anesthetic and implanted at the tibia position and subsequently irradiated for 15 min (0.5 W cm^−2^). After light irradiation (0.5 W cm^−2^), the samples were taken out and soaked in LB medium for 6 h. Subsequently, 20 µL LB medium was used for spread plate in each group and incubated for next 24 h for observing the bacterial colonies. Then, the rats were executed after additional 1 D and 3 D feeding and analyzed through immunofluorescence staining (CD31), TNF‐*α*, IL‐6 of irradiated tissues, and H&E staining of irradiated tissues and main organs (heart, liver, spleen, lungs, and kidney). Meanwhile, whole blood was taken out of the treated rats and detected after 1 D, 2 D, and 3 D.

##### Statistical Analysis

All the data in this work in vitro and in vivo was analyzed through the one way‐analysis of variance (ANOVA) using the software GraphPad prism 7 and performed with *n* ≥ 3 ± SD in every group, and *p < 0.05 represented significance.

## Conflict of Interest

The authors declare no conflict of interest.

## Supporting information

Supporting Information
