# Supplementary material for: Photoelectrons Mediating Angiogenesis and Immunotherapy through Heterojunction Film for Noninvasive Disinfection
Source: Adv Sci (Weinh). 2020 Jul 25;7(17):2000023. doi: 10.1002/advs.202000023 (PMC7507565; doi:10.1002/advs.202000023)
Supplement: Supplementary file 1 — Supporting Information [file ADVS-7-2000023-s001.pdf]

Copyright WILEY-VCH Verlag GmbH & Co. KGaA, 69469 Weinheim, Germany, 2020.

Supporting Information

Photoelectrons Mediating Angiogenesis and Immunotherapy through Heterojunction Film for Noninvasive Disinfection

Yuan Li[1], Xiaomo Xu[2], Xiangmei Liu[2,*], Bo Li[3], Yong Han[3], Yufeng Zheng[4], Da-fu Chen[5], Kelvin Wai Kwok Yeung[6], Zhenduo Cui[1], Zhaoyang Li[1], Yanqin Liang[1], Shengli Zhu[1], Xianbao Wang[2], Shuilin Wu[1,*]

Table S1. The detail information in blood routine examination at 1 D.

parameter	Ti+L	GO/NCDs/Hap/Ti+L	GO/NCDs/Ti+L	unit
RBC	6.231	15.083	19.706	10^{12}/L
HGB	113.0	292.2	378.0	g/l
HCT	39.2	94.7	121.7	%
MCV	65.1	64.6	60.5	fl
MCH	21.7	23.2	21.8	pg
MCHC	312.0	333.7	336.0	g/l
RDW	11.5	11.3	11.1	%
PLT	617.0	969.6	1057.7	10^{9}/L
MPV	4.7	5.9	5.4	fl
PDW	15.2	14.6	14.3	%
PCT	0.243	0.795	0.618	%

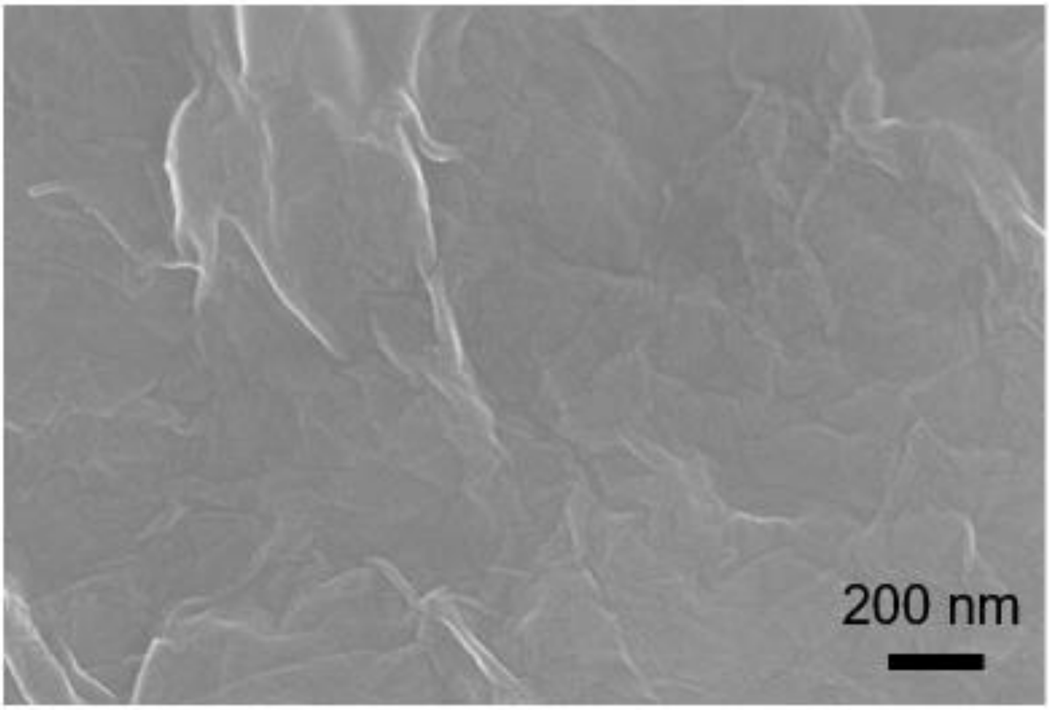

Figure S1. The SEM of GO/NCDs/Ti.

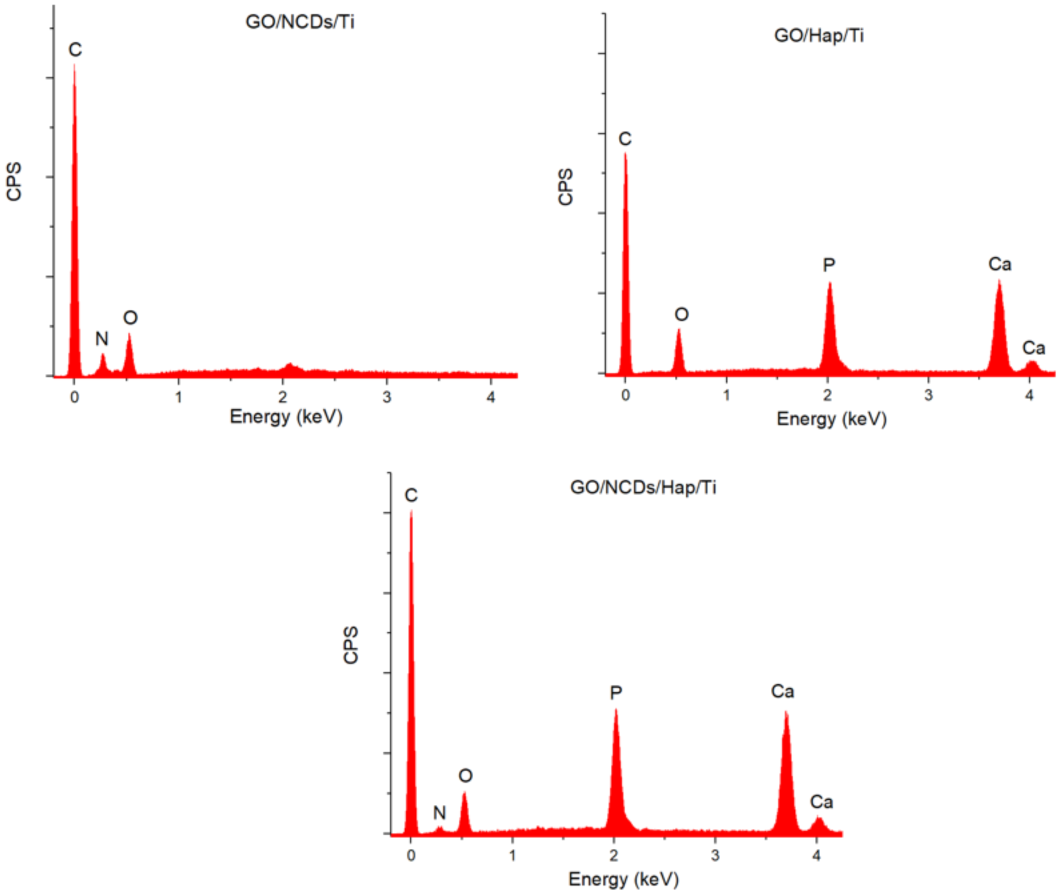

Figure S2. The element EDS of GO/NCDs/Ti, GO/Hap/Ti, and GO/NCDs/Hap/Ti.

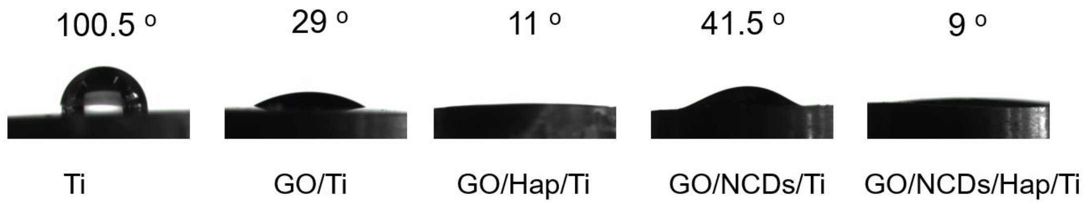

Figure S3. The contact angles of Ti, GO/Ti, GO/NCDs/Ti, GO/Hap/Ti and GO/NCDs/Hap/Ti.

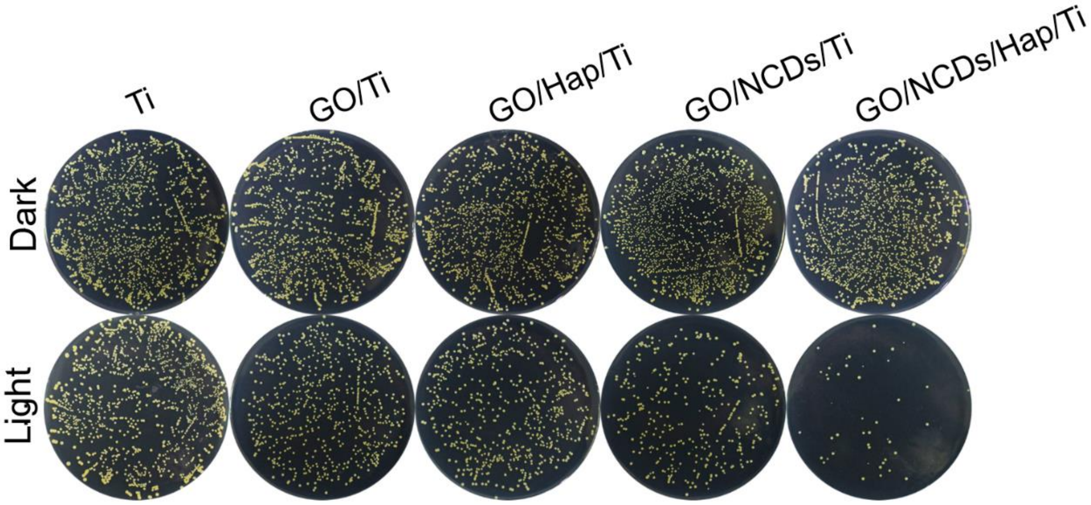

Figure S4. The antibacterial efficiency towards *S. aureus* of Ti, GO/Ti, GO/NCDs/Ti, GO/Hap/Ti and GO/NCDs/Hap/Ti after NIR irradiation for 15 min.

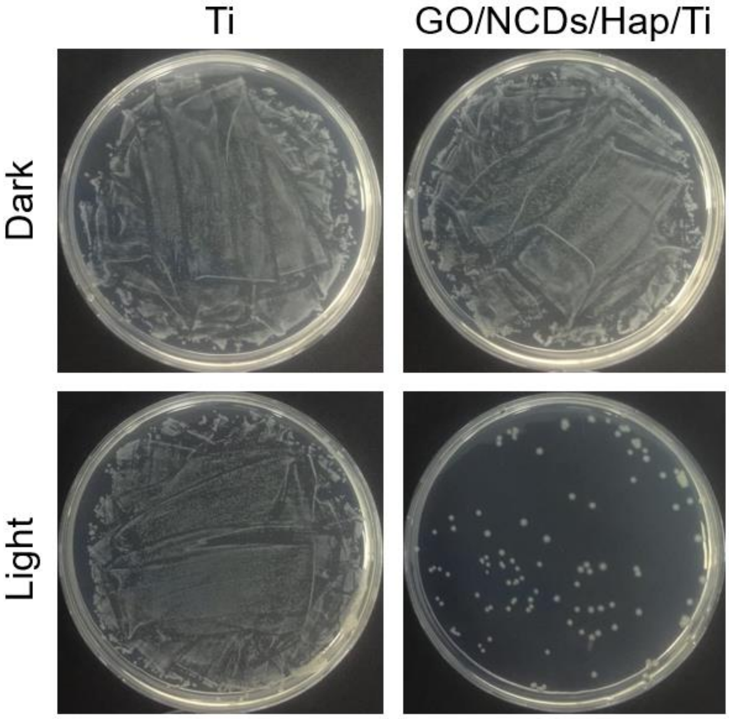

Figure S5. The antibacterial efficiency of Ti, GO/Ti, GO/NCDs/Ti, GO/Hap/Ti and GO/NCDs/Hap/Ti towards *E. coli* after NIR irradiation for 15 min.

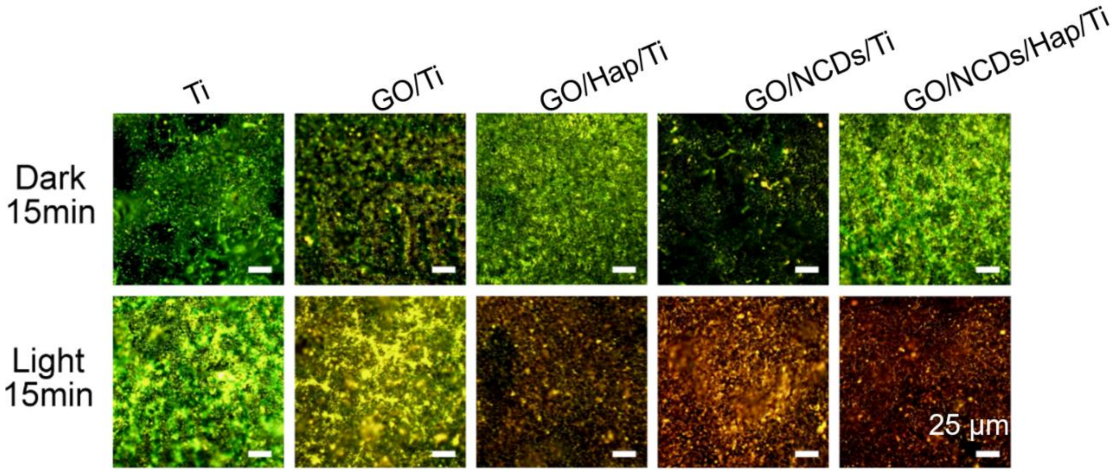

Figure S6. The live-dead fluorescence staining (green indicated live *S. aureus*, red indicated dead *S. aureus*) of Ti, GO/Ti, GO/NCDs/Ti, GO/Hap/Ti and GO/NCDs/Hap/Ti after NIR irradiation for 15 min.

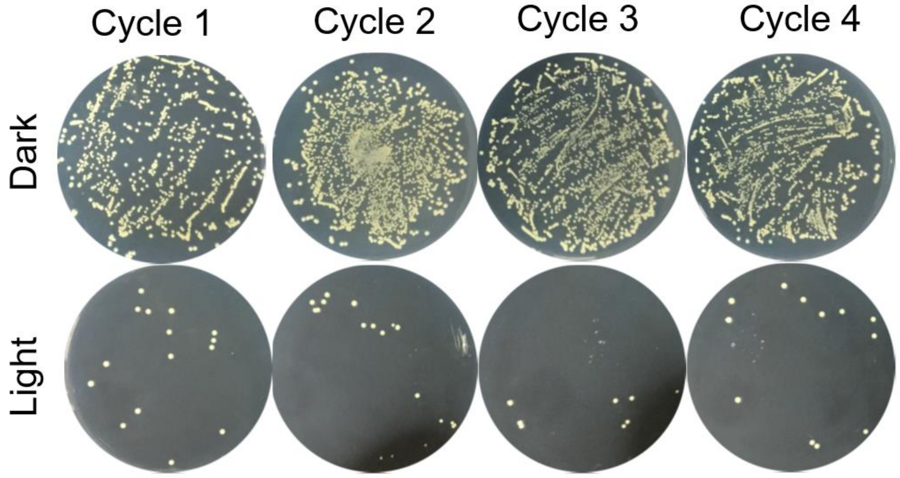

Figure S7. The spread plates of 4 cycles of Ti, GO/Ti, GO/NCDs/Ti, GO/Hap/Ti and GO/NCDs/Hap/Ti after NIR irradiation for 15 min.

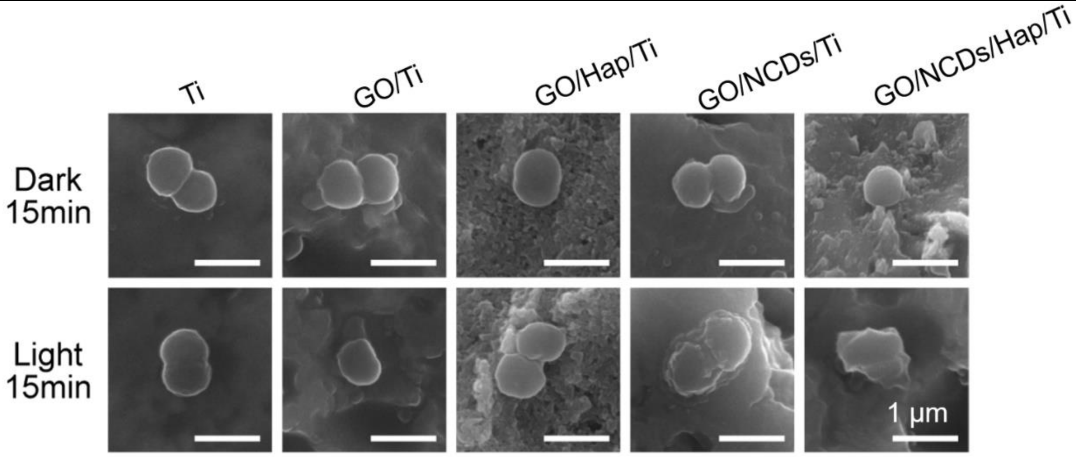

Figure S8. The SEM morphology of *S. aureus* on Ti, GO/Ti, GO/NCDs/Ti, GO/Hap/Ti and GO/NCDs/Hap/Ti after NIR irradiation for 15 min.

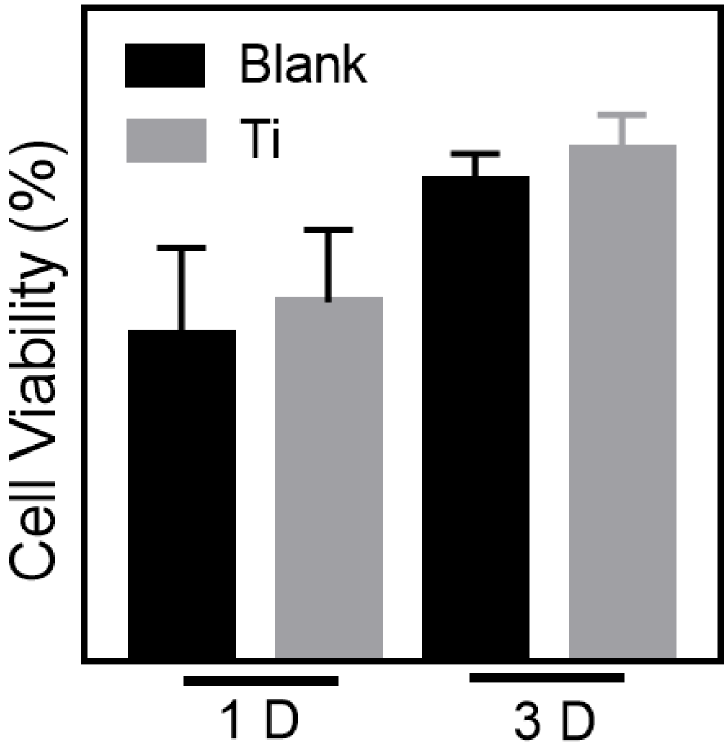

Figure S9. The cell viability of Ti group and the blank group at 1 D and 3 D.

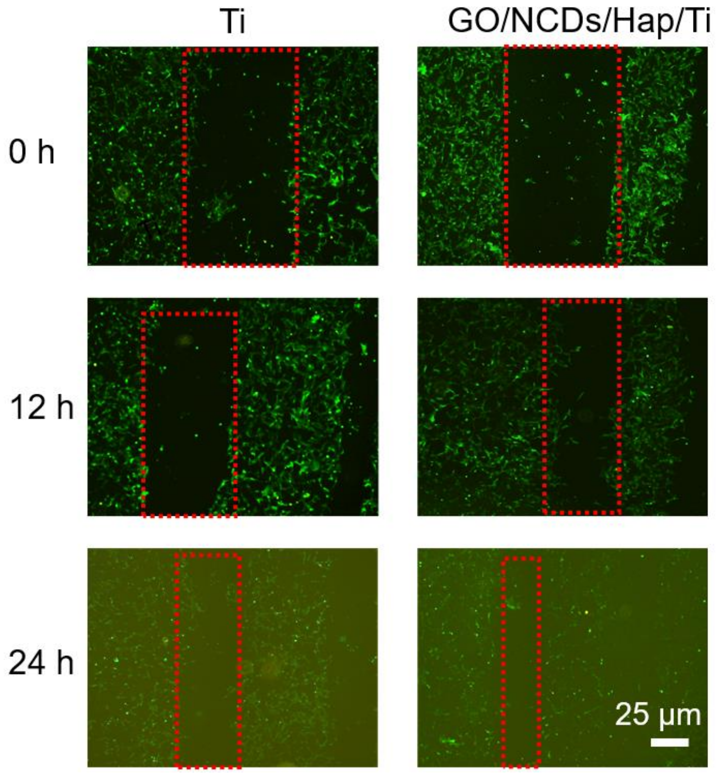

Figure S10. The cells scratch assay of Ti and GO/NCDs/Hap/Ti after NIR irradiation at different incubation time.

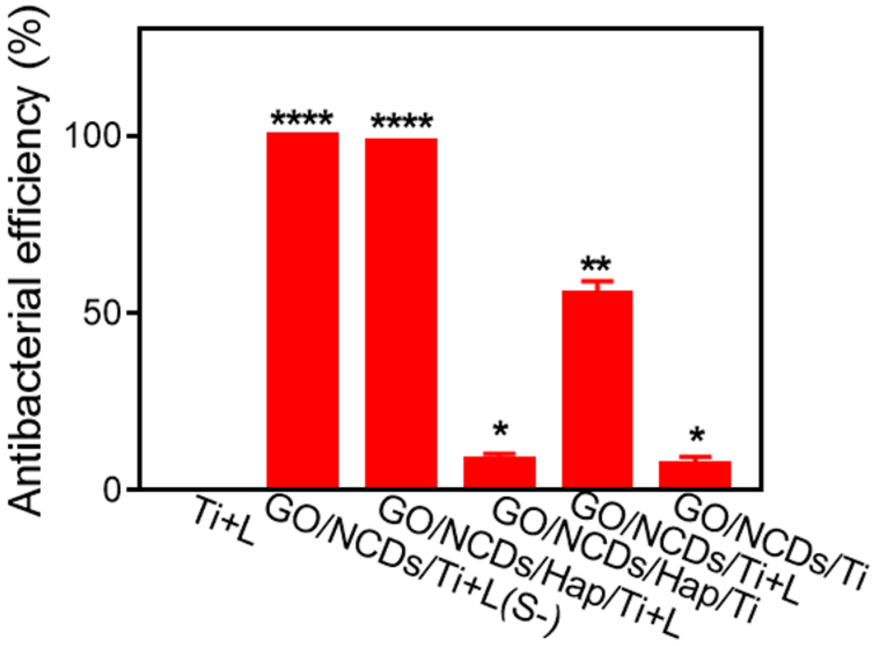

Figure S11. The antibacterial efficiency after NIR irradiation for 15 min *in vivo*.

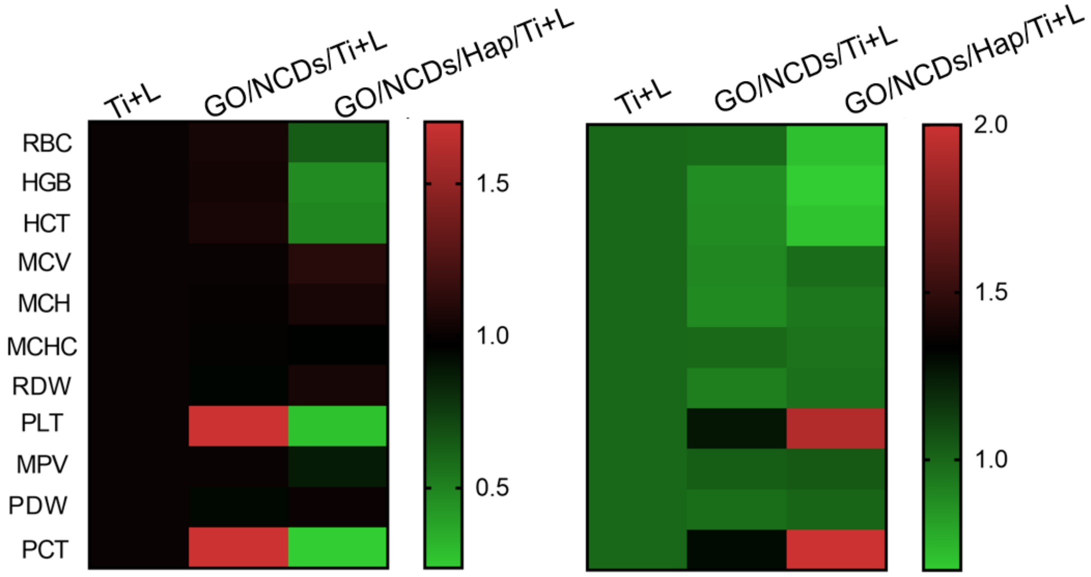

Figure S12. The blood routine examination after NIR irradiation for 15 min at 2 D (left) and 3 D (right) *in vivo*.

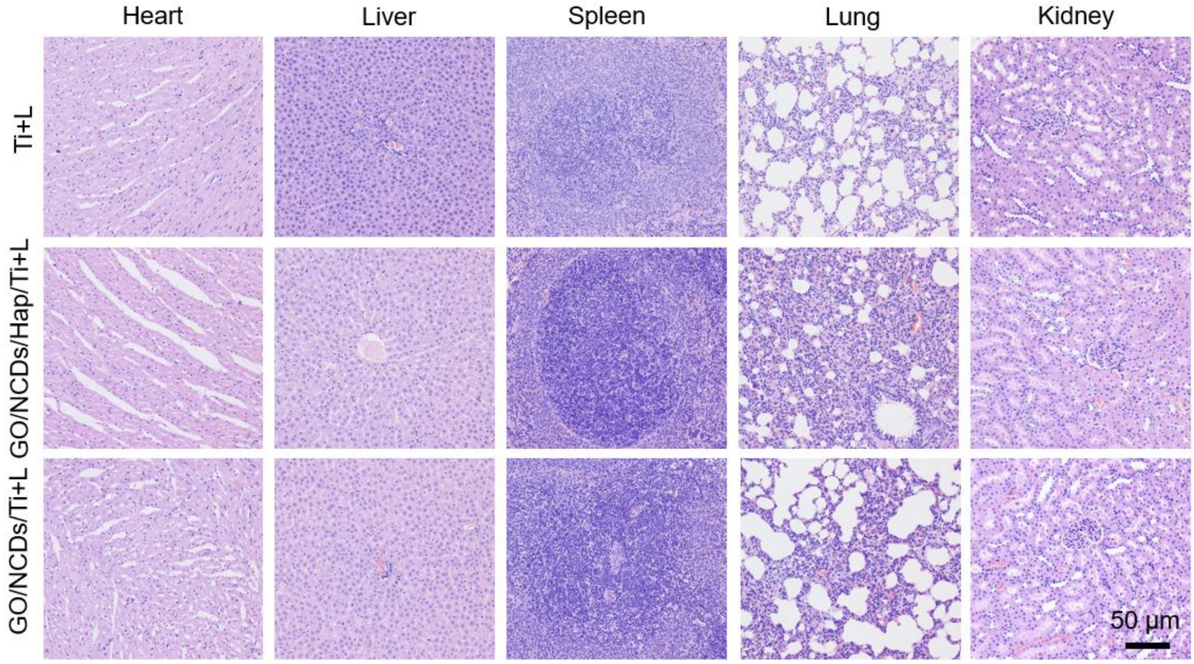

Figure S13. The H&E staining of main organs at 3 D treatment *in vivo*.

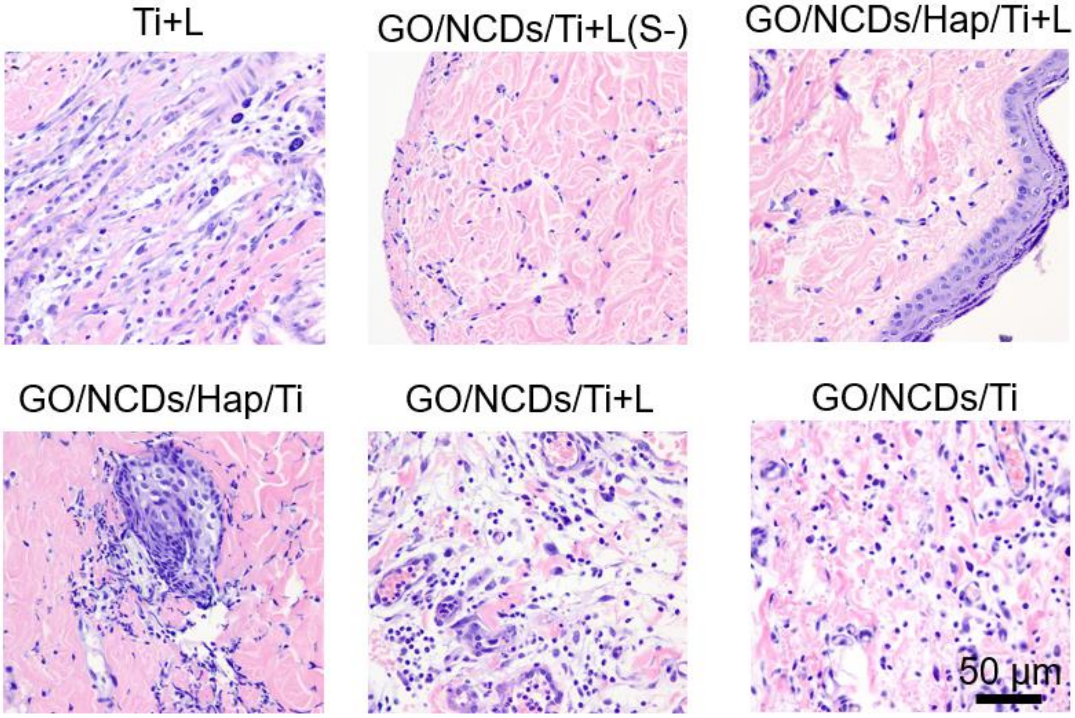

Figure S14. The H&E staining of infected tissues at 3 D treatment *in vivo*.

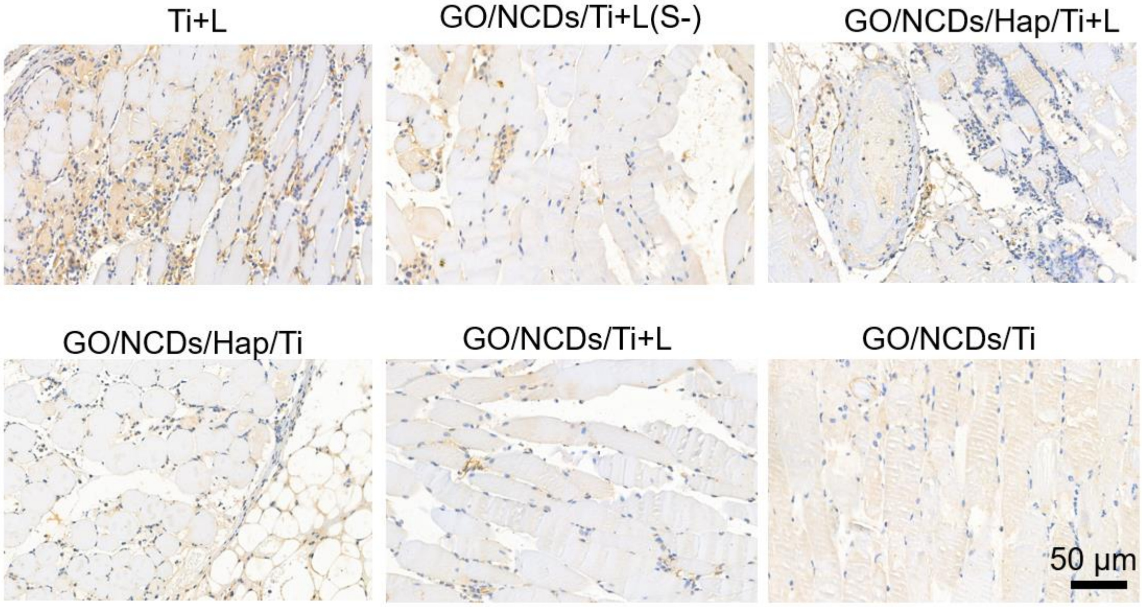

Figure S15. The immunohistochemical of infected tissues at 3 D treatment *in vivo* stained by CD31.

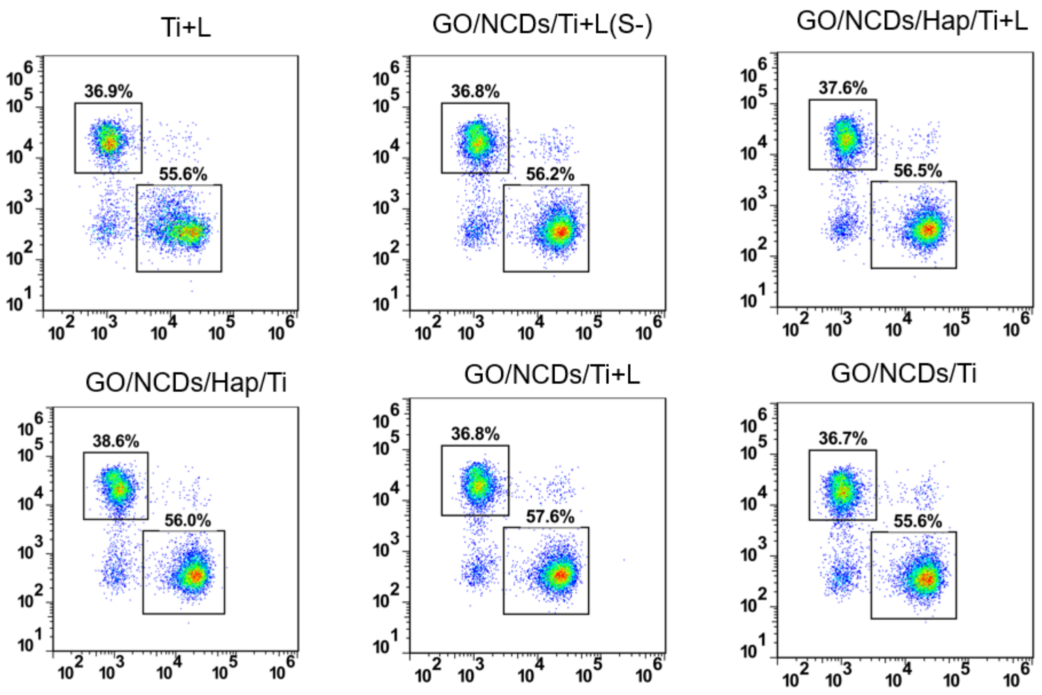

Figure S16. The flow cytometry of CD4 and CD8 lymphocyte in the tissue after irradiation for 3 D.

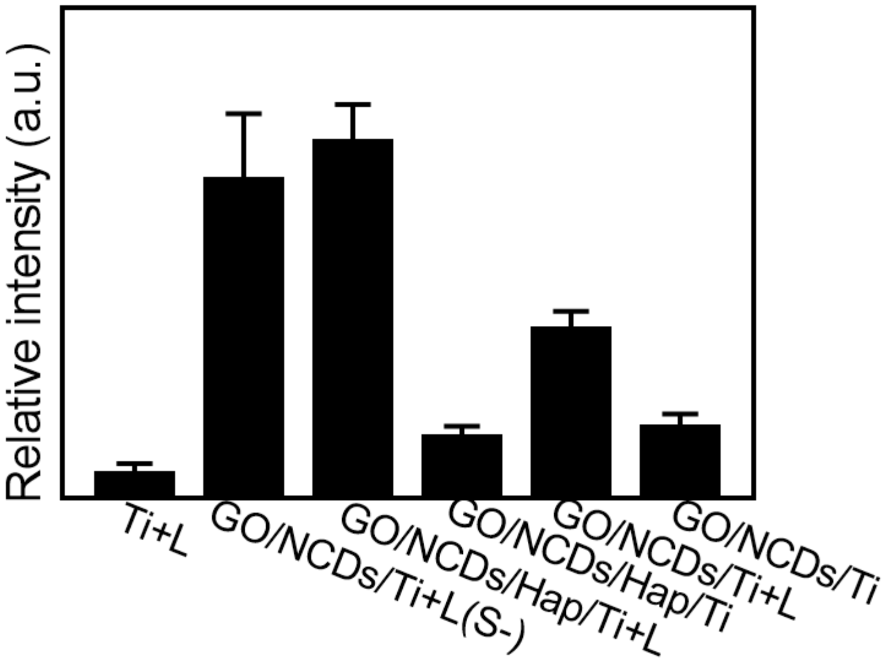

Figure S17. The data about neutrophil granulocyte at 3 D
